# Chronic hepatitis B infection presenting with chronic lymphocytic inflammation with pontine perivascular enhancement responsive to steroids (CLIPPERS): a case report

**DOI:** 10.1186/s13256-015-0750-1

**Published:** 2015-11-19

**Authors:** Ching-Fu Weng, Ding-Cheng Chan, Ya-Fang Chen, Fei-Chih Liu, Horng-Huei Liou

**Affiliations:** 1 Department of Gerontology and Geriatrics, National Taiwan University Hospital, Taipei, Taiwan; 2 Department of Internal Medicine, National Taiwan University Hospital, Taipei, Taiwan; 3 Department of Medical Imaging, National Taiwan University Hospital, Taipei, Taiwan; 4 Department of Neurology, National Taiwan University Hospital, No.1, Changde Street, Zhongzheng District, Taipei City, 10048 Taiwan

**Keywords:** Autoimmunity, CLIPPERS, Demyelinating disease, Encephalomyelitis, Hepatitis B, Regulatory T cell

## Abstract

**Introduction:**

Chronic lymphocytic inflammation with pontine perivascular enhancement responsive to steroids is a brainstem disorder characterized by perivascular pathologic reaction with lymphocyte infiltration and leading to diplopia, facial palsy, dysarthria, and gait ataxia. It was thought to be an autoimmune disorder without distinct pathogenesis. Chronic hepatitis B virus infection has been proposed in correlation with autoimmune diseases, including central nervous system demyelinating disease. Patients with chronic hepatitis B infection may develop the syndrome of chronic lymphocytic inflammation with pontine perivascular enhancement responsive to steroids.

**Case presentation:**

A 34-year-old Taiwanese man who had been a hepatitis B virus carrier for a decade presented to our emergency room. He had influenza symptoms and progressive symptoms of left hemifacial numbness, double vision, and an unsteady gait of 2 days’ duration. Chronic lymphocytic inflammation with pontine perivascular enhancement responsive to steroids was diagnosed, with increased hepatitis B viral load at the same time. He had no past history of similar neurologic deficits, and his liver function tests had been within normal limits before this episode. After corticosteroid and entecavir treatments, his neurological deficits and neuroimaging anomalies improved and his serum hepatitis B virus DNA viral load normalized.

**Conclusions:**

Hepatitis B virus infection may induce central nervous system autoimmune reactions, including chronic lymphocytic inflammation with pontine perivascular enhancement responsive to steroids. In such cases, concomitant administration of corticosteroids and antiviral agent was helpful. We suggest further investigations in patients with regulatory T cell dysfunction, which may assist in clarifying a loss of immune tolerance in patients with such disorders.

## Introduction

Chronic lymphocytic inflammation with pontine perivascular enhancement responsive to steroids (CLIPPERS) is a disorder established since 2010 [1]. Its clinical features include diplopia, facial palsy, dysarthria, and gait ataxia, which result from a brainstem perivascular pathologic reaction with lymphocytic infiltration. Brain magnetic resonance imaging (MRI) reveals punctate and curvilinear gadolinium enhancement in the pons, sometimes with midbrain, cerebellum, spinal cord, or basal ganglia involvement. CLIPPERS responds well to corticosteroid treatment [1]. Even with good improvement in clinical symptoms and resolution observed by neuroimaging after treatment, recurrence or relapse may develop after discontinuing or tapering corticosteroid therapy. The distinct pathophysiology of CLIPPERS is still unknown. Several studies suggested CLIPPERS as an autoimmune disorder, but the hypothesis is still not well established, owing to a paucity of data [1, 2]. In this report, we describe a case of a patient with a clinical diagnosis of CLIPPERS. He was a chronic hepatitis B virus (HBV) carrier with elevated HBV DNA viral load. His presentation raised the possibility of HBV-associated immunity and regulatory T cell (Treg) dysfunction as the cause of the pathogenesis of CLIPPERS. To our knowledge, this is the first case of CLIPPERS associated with the chronic HBV infection reported to date.

## Case presentation

A 34-year-old Taiwanese man was a known HBV carrier for about 10 years. Headache, lightheadedness, neck stiffness, and nausea and vomiting had developed 3 weeks before admission. One week after admission, left hemifacial numbness, double vision, easily choking when swallowing, frequent hiccup, and unsteady gait developed and progressed.

The patient had no cough, rhinorrhea, abdominal pain, diarrhea, dysuria, urinary frequency, convulsive movement, consciousness disturbance, or body weight loss. He sought Chinese traditional medical therapy and acupuncture, but his neurologic symptoms persisted, especially the easily choking when swallowing. Therefore, he presented to our emergency room.

**Fig. 1:**
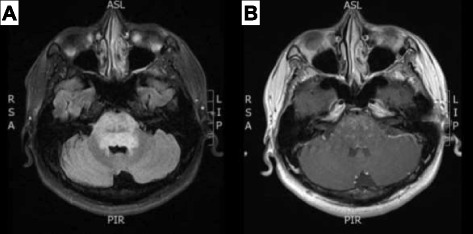
**a** Fluid-attenuated inversion recovery image shows ill-defined hyperintensity involving the whole pons, which was associated with swelling of the pons. **b** Postcontrast T1-weighted image shows multiple punctate and curvilinear enhancing lesions in the pons bilaterally

Upon admission, the patient’s initial blood pressure was 126/76 mmHg and his temperature was 37.3 °C. His pulse rate was 68 beats/minute. A neurological examination revealed impaired lateral gaze of both eyes and mild deviation of the tongue to the left side. Cerebellar dysarthria, wide-based gait ataxia, and bilateral upper limb dysmetria were detected. The patient’s brain MRI showed swelling of the pons with extensive hyperintensity on T2-weighted images that extended to the midbrain and medulla. Punctate and curvilinear enhancement was observed after contrast dye injection (Fig. 1). A cerebrospinal fluid (CSF) study disclosed pleocytosis (106 cells/μl) with lymphocyte predominance (lymphocytes/neutrophils 97%/9%). Oligoclonal bands were detected (total protein 79.9 mg/dl, normal 15–45 mg/dl; immunoglobulin G (IgG) 9.72 mg/dl, normal <3.4 mg/dl). Test results for herpes simplex virus DNA and cryptococcal antigen in the CSF were both negative. Serial autoimmune serum examination results, including antinuclear antibodies, anti-DNA, cytoplasmic antineutrophil cytoplasmic antibodies, perinuclear antineutrophil cytoplasmic antibodies, rheumatoid factor, complement C3 and C4 nephritic factors, IgM, IgG, were all within normal limits, except for a slightly elevated IgA level (409 mg/dl, normal 70–400 mg/dl). The patient’s anti-hepatitis B e (anti-HBe) antibody test was positive. His total bilirubin was 0.56 mg/dl, and his aspartate aminotransferase (AST) concentration was 24 U/L. An elevated HBV DNA viral load (2250 IU/ml, normal <20 IU/mL) was detected.

**Fig. 2:**
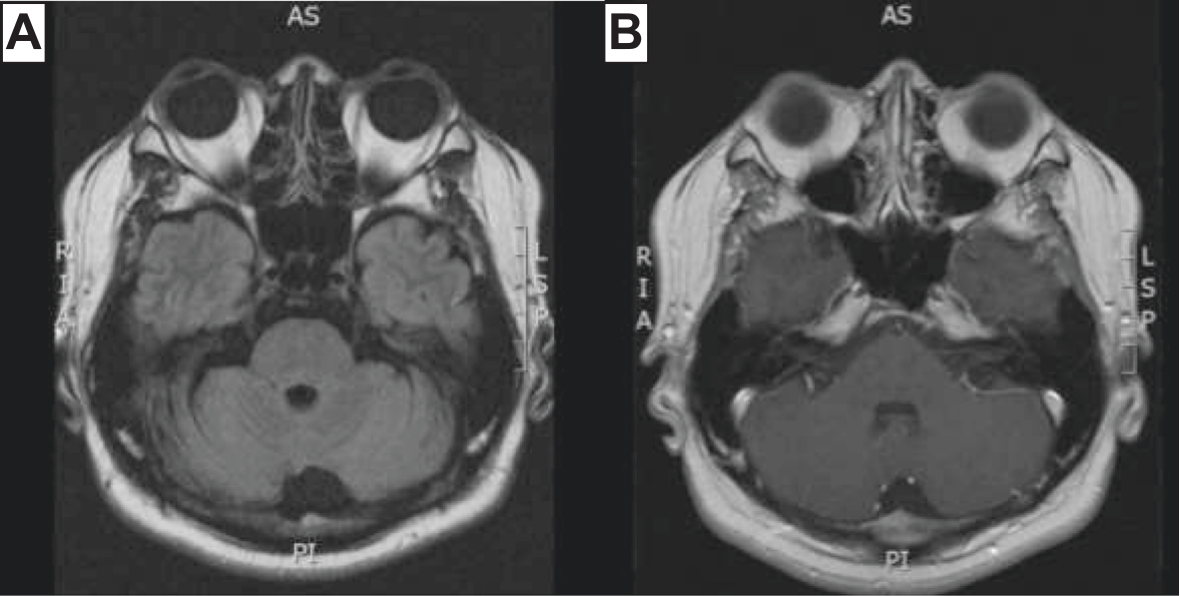
Images obtained 6 months after corticosteroid treatment. **a** Fluid-attenuated inversion recovery image. **b** Postcontrast T1-weighted image showing nearly complete resolution of the patient’s pontine lesions

Corticosteroid therapy was considered based on a tentative diagnosis of CLIPPERS. Entecavir 1 mg/day was given before a high-dose corticosteroid (1 g/day) after consultation with a gastroenterologist. A maintenance dose of 0.5 mg/day was started subsequently, followed by prednisolone 70 mg/day. The patient’s bilateral lateral gaze limitation, unsteady gait, dysarthria, and dysphagia improved promptly. The patient was discharged 3 weeks later with a good clinical response, and treatment was continued with gradual tapering of prednisolone to 30 mg/day over the course of 6 months. Six months later, nearly complete resolution of the brainstem lesion was seen on MRI scans (Fig. 2). Normalization of the patient’s serum HBV DNA viral load (<20 IU/mL) and disappearance of his neurologic deficit also were noted.

## Discussion

Our patient had several clinical and typical radiologic findings that met the diagnostic criteria for CLIPPERS, although the tissue proof was not acquisitive. The diagnosis of CLIPPERS is generally based on clinical, radiological, and pathological findings and glucocorticoid response. Brain biopsy in critical areas of the brainstem has potential complications. A clinical diagnosis based on typical image appearance is crucial [3], but clinicians should still be aware of a constellation of various presentations of CLIPPERS. Cautious exclusion of similar central nervous system (CNS) inflammation disorders is mandatory.

The differential diagnosis should include Bickerstaff’s brainstem encephalitis, CNS vasculitis (primary angiitis of the CNS), neuromyelitis optica, Sjögren’s syndrome with CNS involvement, CNS lymphoma, and glioma (especially when atypical clinical or MRI findings are encountered), or when resistance to corticosteroid treatment occurs [4]. Once the diagnosis is made, the treatment should be started immediately to achieve an optimal outcome, even if a biopsy is not performed for pathologic proof.

Investigators proposed that this disorder is an autoimmune process involving perivascular inflammatory reaction, but the exact mechanism remains unknown. Kastrup and colleagues described two cases of CLIPPERS with elevated IgE level, and eosinophilic granulocytes were absent in both cases [2]. IgE-related neuroinflammation was considered [5]. Influenza vaccination–induced CLIPPERS has been reported as well [6]. Nevertheless, such hypersensitivity reactions may not fully explain the pathogenesis of CLIPPERS. Furthermore, there is still no specific antineural surface antigen antibody that can be recognized [7].

Our patient had been diagnosed as a chronic HBV carrier for more than 10 years. He had undergone regular abdominal ultrasonography imaging and serum liver function tests during that time. The liver echotexture and serum biomarkers were all within normal limits. Before corticosteroid administration after his admission to our hospital, elevated HBV DNA viral load (2250 IU/ml) was detected. His serum HBe antigen was negative, his anti-HBe antibody was positive, and his AST and alanine aminotransferase levels were within normal limits. With high-dose corticosteroid use, a flare-up of his HBV infection was possible; thus, adding antiviral therapy was a reasonable choice from both therapeutic and prevention standpoints [8].

HBV infection has been proposed in correlation with autoimmune diseases, such as multiple sclerosis (MS), systemic lupus erythematosus, rheumatoid arthritis, polymyalgia rheumatica, polymyositis, and autoimmune liver disease. Several possible mechanisms have been postulated: molecular mimicry, epitope spreading and modification, viral or bacterial superantigens, release of autoantigens during inflammation, or bystander activation [9–12]. For example, investigators who used an animal model of MS experimental autoimmune encephalomyelitis showed that rabbit T cells were reactive to both injected HBV polymerase peptide and myelin basic protein at the same amino acid, resulting in autoimmune demyelinating encephalomyelitis [13]. In patients with autoimmune liver disease caused by occult HBV infection, isolated serum HBV DNA was also detected in significant proportions [14].

Xu and colleagues pointed that high serum HBV DNA viral load has a positive correlation with circulating CD4^+^CD25^+^Treg frequency in peripheral blood, suggesting that an increase in Tregs correlates with impairment in viral clearance [15], leading to persistence of HBV infection [16]. Tregs are able to maintain immunological homeostasis and suppress autoimmunity against self-antigens [17]. Impaired Treg function in peripheral blood caused by complex genetic defects or environmental factors may play a role in HBV-related, cell-mediated autoimmunity and may have contributed to the increased HBV DNA viral load in our patient.

We suggest adding anti-HBV therapy to steroid therapy and monitoring of HBV viral activity in individuals diagnosed with CLIPPERS concomitant with chronic HBV infection. Further surveys of the role of Tregs in CLIPPERS may lead to a better understanding of the pathogenesis of this disorder and help clinicians to find a new strategy in developing more effective therapy.

## Conclusions

Increasing numbers of reports have recently indicated that CLIPPERS is an emerging entity associated with an autoimmunologic process involving the CNS. To the best of our knowledge, we report the first case associated with HBV infection. In our patient, the neurologic manifestation correlated well with HBV viral load. Long-term corticosteroid use had been recommended for CLIPPERS because of the easily relapsing-remitting character of the disease. When this disorder is accompanied by chronic HBV infection, antiviral therapy is strongly recommended. Thus, we assumed that dual use of an antiviral agent and a steroid for suppression of virus-associated autoimmune reaction is beneficial for treatment. Further investigation of Tregs in CLIPPERS may help to clarify the disease process.

## Consent

Written informed consent was obtained from the patient for publication of this case report and any accompanying images. A copy of the written consent is available for review by the Editor-in-Chief of this journal.

## Abbreviations

AST: aspartate aminotransferase
CLIPPERS: chronic lymphocytic inflammation with pontine perivascular enhancement responsive to steroids
CNS: central nervous system
CSF: cerebrospinal fluid
HBe: hepatitis B e
HBV: hepatitis B virus
Ig: immunoglobulin
MRI: magnetic resonance imaging
MS: multiple sclerosis
Treg: regulatory T cell
